# Trends in research related to ophthalmic microperimetry from 1992 to 2022: A bibliometric analysis and knowledge graph study

**DOI:** 10.3389/fmed.2022.1024336

**Published:** 2023-01-19

**Authors:** Jing Ming, Rui Qin

**Affiliations:** ^1^Xiyuan Hospital, China Academy of Chinese Medical Sciences, Beijing, China; ^2^Department of Ophthalmology, Eye Hospital, China Academy of Chinese Medical Sciences, Beijing, China

**Keywords:** bibliometric analysis, microperimetry, VOSviewer, CiteSpace, keyword, journal

## Abstract

**Background:**

Microperimetry is a novel technology to assess macular function. The aim of the study was to explore the global research status and trends in microperimetry.

**Methods:**

Documents related to microperimetry in ophthalmology from 1992 to 2022 were extracted from the Science Citation Index Expanded (SCI-E) database of the Web of Science Core Collection (WOSCC). Raw data were analyzed using the VOSviewer and CiteSpace software. Bibliometric parameters included annual publication quantity, countries, authors, journals, international cooperation, and keywords.

**Results:**

A total of 1,217 peer-reviewed documents were retrieved. Annual research output has increased significantly since 2005, especially since 2013. Holz F, Rubin G, and Guymer R contributed most to the number of articles published about microperimetry. Rubin G, Fitzke F, and Holz F, respectively, received the most citations for their study. The countries publishing most were the USA, Italy, and the UK, while the USA, the UK, and Germany received the most citation frequency. Univ Bonn, UCL, and Moorfields Eye Hosp were the top three productive institutions for microperimetry research in the world. The top three journals that publish articles about microperimetry were Retina-The Journal of Retinal and Vitreous Diseases, Investigative Ophthalmology and Visual Science, and the American Journal of Ophthalmology. The top 10 common keywords included microperimetry, optical coherence tomography, eye, retinal sensitivity, macular degeneration, fundus autofluorescence, scanning laser ophthalmoscope, visual acuity, sensitivity, and degeneration. Keywords “optical coherence tomography angiography,” “retinitis pigmentosa,” and “internal limiting membrane” burst in the last 3 years.

**Conclusion:**

The bibliometric and knowledge graph analysis of research status and trends in microperimetry provided global researchers with valuable information to propose future cooperation and track cutting-edge progress.

## 1. Introduction

In recent years, the development of retinal imaging techniques brought revolutionary changes to the diagnosis and prognosis evaluation of retinal diseases. However, subtle lesions are challenging to detect in morphological tests. In addition, similar changes in the retina might lead to very different outcomes. Therefore, functional testing is required to solve the shortage of morphological tests. Routine perimetry examination is practical for peripheral retina or optic neuropathy, e.g., glaucoma (1) and retinitis pigmentosa (2). Nevertheless, in the macular function assessment, the standard perimetry test shows the limitations of high test–retest variability (3). Microperimetry is a diagnostic method that combines psychophysical methods and fundus imaging techniques to assess the severity of macular diseases comprehensively. Microperimetry projects stimuli directly on retinal regions of interest, as opposed to standard perimetry which projects stimuli on a spherical cupula that are back-reflected. This feature enables continuous real-time tracking during the course of microperimetric examination, which can help to minimize the measurement noise during the course of the psychophysical assessment. Besides, it has a particular advantage in mapping visual function defects to the particular location of the retina (4).

Microperimetry was widely utilized in the field of central serous chorioretinopathy (5), central retinal vein occlusion (6), branch retinal vein occlusion (7), diabetic retinopathy (8), macular degeneration (9), etc.

With the help of bibliometrics and knowledge graphs, we were allowed to analyze the research status and trends in microperimetry. Co-authorship and co-occurrence network analysis were important components of bibliometric methods. Apart from this, citation analysis was used to evaluate the importance and academic value of research. The bibliometric analysis collected the articles on microperimetry over the past 30 years to identify the authors, research groups, and countries clustering. Then the hotspot of the field was analyzed by keyword analysis.

## 2. Materials and methods

### 2.1. Database selection and search strategy

The relevant literature was retrieved from the Science Citation Index Expanded (SCI-E) database of the Web of Science Core Collection (WOSCC) on 21 July 2022. The search formula was TS = (microperimetry OR microperimeter OR microperimetric OR microperimeters).

### 2.2. Inclusion and exclusion criteria

The search results were limited by language (English) and the publication year (1992–2022). The excluded literature was as follows: meeting abstracts (214), proceeding papers (48), letters (23), early access (16), and editorial materials (7).

### 2.3. Statistical analysis and visualization methods

Graphpad Prism (v9.3.1) was utilized to visualize the descriptive statistics. VOSviewer (v1.6.18.0) was enrolled to analyze top countries, journals, author affiliations, and keywords. In addition, CiteSpace (v6.1.R2) was used to analyze co-cited references and research trends. All data were downloaded from the WOSCC database as secondary data, with no further animal trials. As a result, the study was exempt from ethical approval. Furthermore, countries-authors-journals plot and wordcloud were generated by the R package “Bibliometrix” (ver 4.0.0) (10).

The geographical map was generated by the “rworldmap” package (v1.3–6) of the R platform (v4.1.1).

**FIGURE 1:**
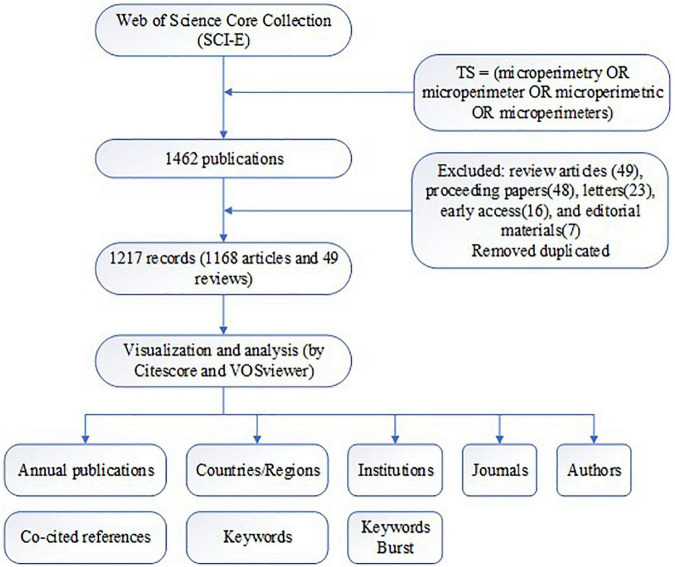
Workflow of the study.

The workflow of the research is presented below (Figure 1).

## 3. Results

### 3.1. Annual trends of publications

**FIGURE 2:**
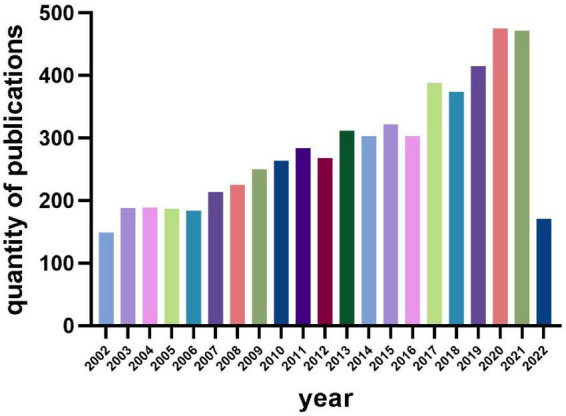
Annual publication quantity.

**FIGURE 3:**
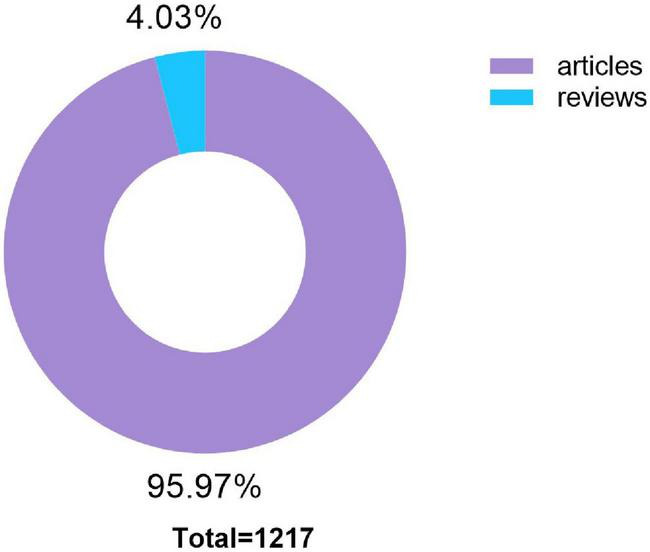
Distribution of publication types.

There were a total of 1,217 microperimetry-related articles included in the research after removing duplication. A histogram was built to visualize the annual research trends in microperimetry (Figure 2). It showed the research on microperimetry developed rapidly from 2005 and soared in 2013. Original articles accounted for 95.97% of document type (Figure 3), suggesting that microperimetry was an emerging research field that was not systematic enough.

### 3.2. Author analysis

**FIGURE 4:**
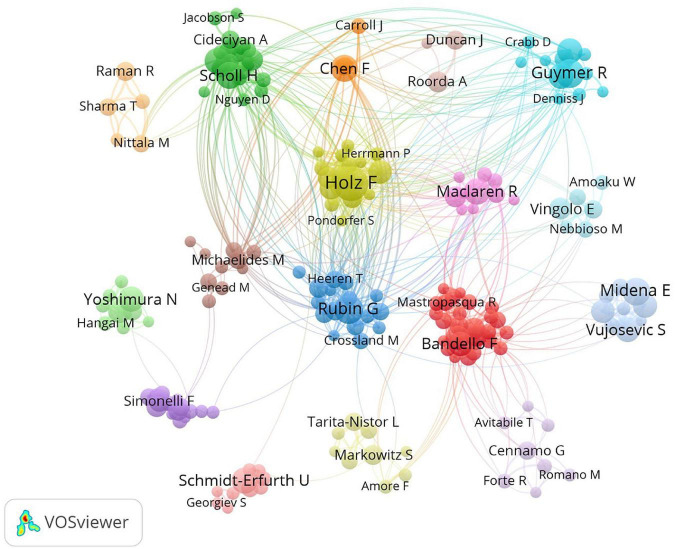
Co-authorship map of authors. The size of nodes demonstrated the frequency of authorship. The curves between the nodes illustrated their co-authorship in the same publication. The shorter distance between the two nodes showed the closeness of the co-authorship of the two authors.

The productive and impactful authors are listed in Tables 1, 2 and visualized in Figure 4. The results showed that Holz F, Rubin G, and Guymer R contributed the most to the field of microperimetry (Table 1). Rubin G, Fitzke F, and Holz F, respectively, had the most citations for their study (Table 2).

**TABLE 1:** The top 10 authors in the field of microperimetry (rank by documents).

Rank	Author	Documents	Citations	Total link strength
1	Holz F	44	1344	200
2	Rubin G	30	2590	107
3	Guymer R	30	711	90
4	Midena E	29	1148	99
5	Chen F	27	356	112
6	Scholl H	26	849	138
7	Sadda S	24	662	100
8	Maclaren R	24	1305	61
9	Schmidt-Erfurth U	24	568	44
10	Wu Z	23	580	73

**TABLE 2:** The top 10 authors in the field of microperimetry (rank by citations).

Rank	Author	Documents	Citations	Total link strength
1	Rubin G	30	2590	107
2	Fitzke F	5	1647	19
3	Holz F	44	1344	200
4	Maclaren R	24	1305	61
5	Midena E	29	1148	99
6	Vujosevic S	22	1108	75
7	Pilotto E	21	853	75
8	Scholl H	26	849	138
9	Chew E	14	820	49
10	Convento E	20	769	78

### 3.3. Active countries/regions analysis

**FIGURE 5:**
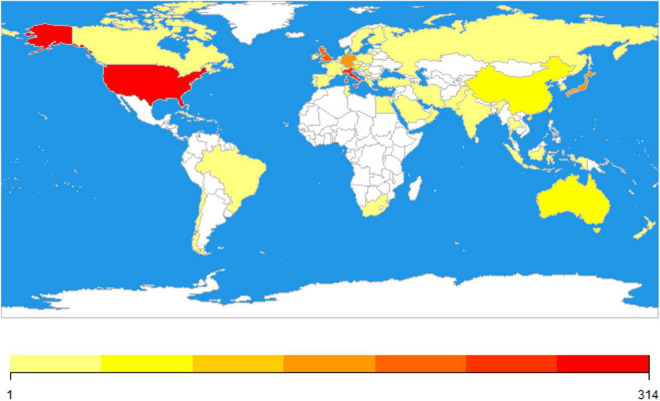
The world map of active countries. Color represented the number of publications. White represented no publication, while from yellow to red illustrated the number of articles. The number of over 314 articles was uniformly expressed in dark red.

**FIGURE 6:**
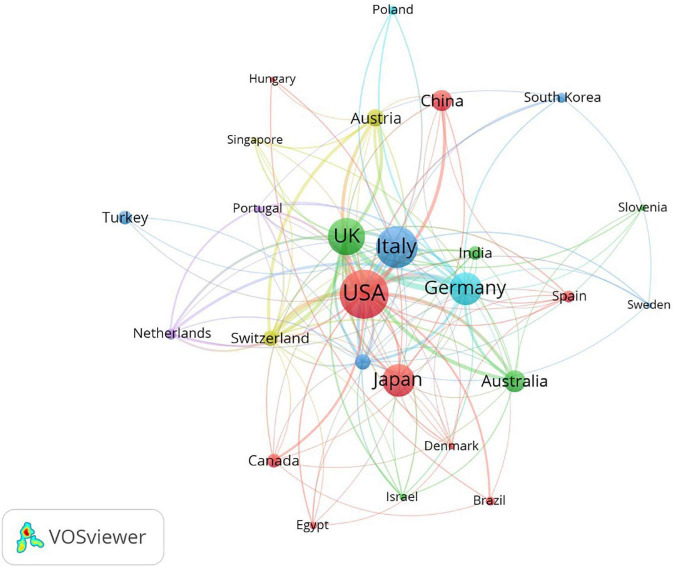
The co-authorship analysis of countries involved in microperimetry research. The size of nodes demonstrated the number of publications by country. The curves between the nodes illustrated their co-operation. The shorter distance between the two nodes indicates a closer level of co-operation between the two countries.

The most active countries/regions are listed in Tables 3, 4. England, Scotland, Wales, and North Ireland were combined into the UK. It showed that the USA, Italy, and the UK contributed the most to the number of articles about microperimetry, while the USA, the UK, and Germany received the most citation frequency. Geographic distribution is visualized in Figure 5. Furthermore, the cooperative condition of countries is visualized in Figure 6.

**TABLE 3:** The top 10 countries contributed to microperimetry research (rank by the number of articles).

Rank	Country	Amount of articles	Citations
1	USA	314	10,648
2	Italy	231	4,202
3	UK	190	6,432
4	Japan	154	2,796
5	Germany	152	4,819
6	Australia	74	1,603
7	China	70	613
8	Austria	52	1,121
9	France	42	1,149
10	Switzerland	41	845

**TABLE 4:** The top 10 countries contributed to microperimetry research (rank by citations).

Rank	Country	Amount of articles	Citations
1	USA	314	10,648
2	UK	190	6,432
3	Germany	152	4,819
4	Italy	231	4,202
5	Japan	154	2,796
6	Australia	74	1,603
7	France	42	1,149
8	Austria	52	1,121
9	Netherlands	23	1,109
10	Canada	36	1,035

### 3.4. Active institutions research

**FIGURE 7:**
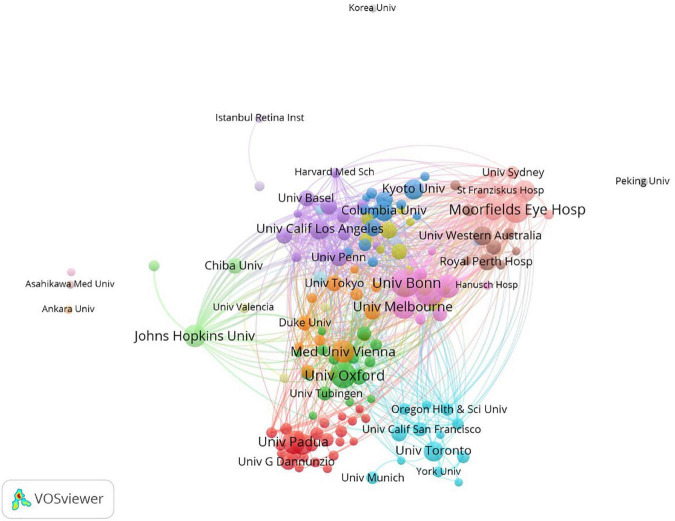
The co-authorship of institutions. The size of nodes demonstrated the number of publications by institutions. The curves between the nodes illustrated their co-operation. The shorter the distance between two nodes showed the closeness of co-operation between the two institutions.

There were many institutions contributing to the microperimetry research. In Table 5, the top 10 productive institutions were listed. Co-authorship analysis is shown in Figure 7. Univ Bonn, UCL, and Moorfields Eye Hosp were the top three productive institutions for microperimetry research in the world. Half of the top 10 most productive institutions were located in the UK.

**TABLE 5:** The top 10 most productive institutions.

Rank	Institutions	Country	Publication
1	Univ Bonn	Germany	52
2	UCL	UK	50
3	Moorfields Eye Hosp	UK	46
4	Univ Oxford	UK	43
5	Univ Padua	Italy	36
6	Moorfields Eye Hosp NHS Fdn Trust	UK	34
7	Univ Melbourne	Australia	34
8	Johns Hopkins Univ	USA	33
9	Med Univ Vienna	Austria	32
10	UCL Inst Ophthalmol	UK	31

### 3.5. Journals research

**FIGURE 8:**
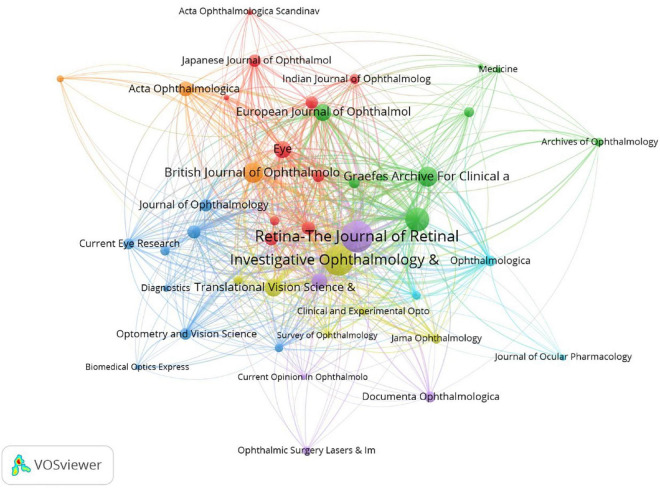
Journal analysis. The size of nodes demonstrated the number of publications by journals. The curves between the nodes illustrated the citation relationship. The color showed the clustering of journals.

Journals publishing most articles about microperimetry are visualized in Table 6 and Figure 8. Retina-The Journal of Retinal and Vitreous Diseases, Investigative Ophthalmology and Visual Science, and American Journal of Ophthalmology were the top three journals to publish articles about microperimetry.

**TABLE 6:** Top 10 journals of microperimetry research.

Rank	Journals	Articles	Cites	IF (2022)	Country
1	Retina-The Journal of Retinal and Vitreous Diseases	143	3104	3.975	USA
2	Investigative Ophthalmology and Visual Science	131	4302	4.925	USA
3	American Journal of Ophthalmology	81	3268	5.488	USA
4	Graefes Archive For Clinical and Experimental Ophthalmology	59	1220	3.535	USA
5	British Journal of Ophthalmology	57	1494	5.908	UK
6	Translational Vision Science and Technology	47	371	3.048	USA
7	Eye	43	1062	4.456	UK
8	European Journal of Ophthalmology	40	492	1.922	Italy
9	Ophthalmology	33	2230	14.277	USA
10	Acta Ophthalmological	30	401	3.988	Denmark

### 3.6. Keyword research

**FIGURE 9:**
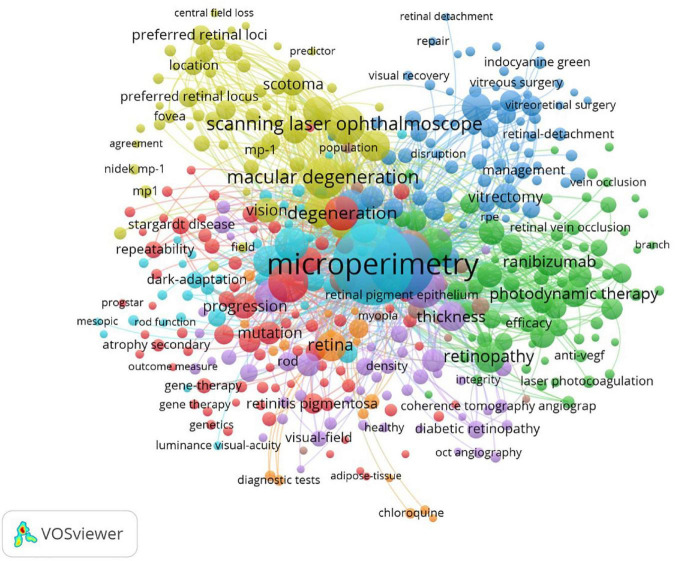
Keyword analysis. The size of nodes demonstrated the number of occurrences of keywords. The curves between the nodes illustrated the co-occurrence between the two keywords. The color showed the clustering of keywords.

Keyword research collected the critical features of the articles. As Table 7 and Figure 9 showed, the top 10 common keywords included microperimetry, optical coherence tomography, eye, retinal sensitivity, macular degeneration, fundus autofluorescence, scanning laser ophthalmoscope, visual acuity, sensitivity, and degeneration.

**TABLE 7:** Top 10 common keywords of microperimetry analysis.

Rank	Keywords	Frequency
1	Microperimetry	593
2	Optical coherence tomography	364
3	Eye	161
4	Retinal sensitivity	135
5	Macular degeneration	133
6	Fundus autofluorescence	127
7	Scanning laser ophthalmoscope	126
8	Visual-acuity	114
9	Sensitivity	113
10	Degeneration	97

### 3.7. Relationship among authors, countries, and journals

**FIGURE 10:**
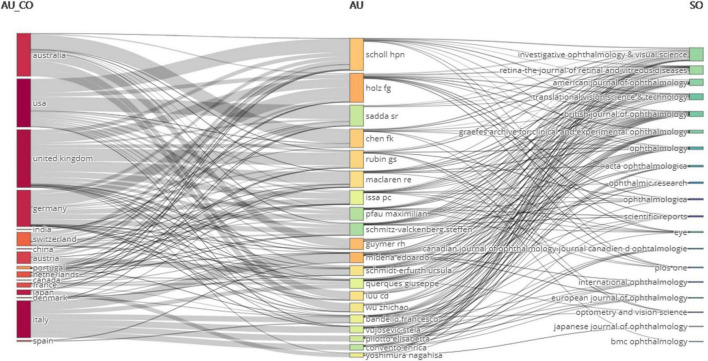
Relationship among authors, countries, and journals. **(Left block)** countries; **(middle block)** authors; **(right block)** journals. Curve linking them demonstrated the relationship among authors, countries, and journals.

Figure 10 shows the relationship among authors, countries, and journals. It implied that the core authors contributed most to the top journals.

### 3.8. Research trends

**FIGURE 11:**
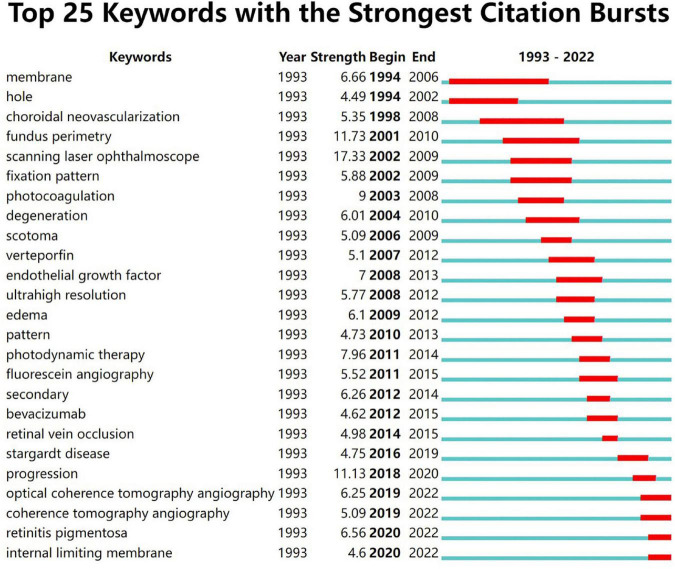
Top 25 keywords with the strongest citation bursts in microperimetry. The red bar in the green timeline demonstrated the burst period of specific keywords.

**FIGURE 12:**
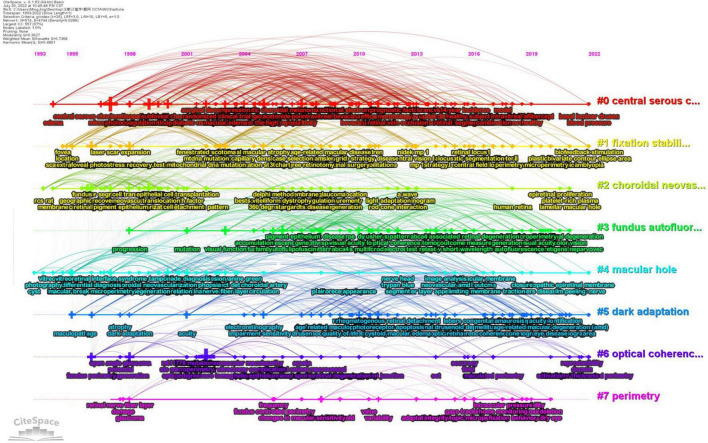
Keyword evolution by timeline. Seven core keywords were arranged as forms of timelines vertically. #0: central serous chorioretinopathy; #1: fixation stability; #2: choroidal neovascularization; #3: fundus autofluorescence; #4: macular hole; #5: dark adaption; #6: optical coherence tomography; #7: perimetry. Each timeline covered a series of keywords.

**FIGURE 13:**
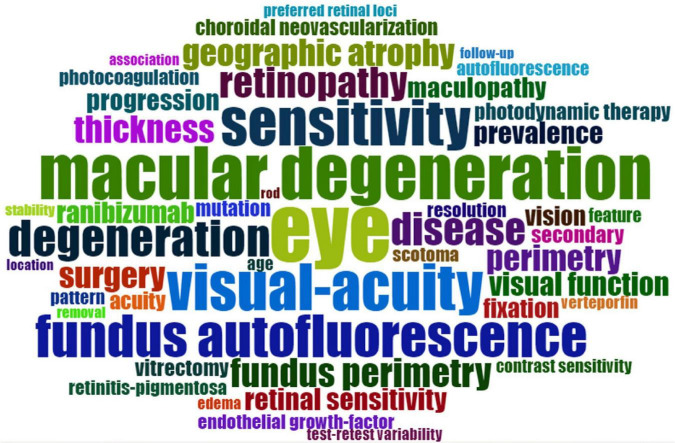
Wordcloud of microperimetry research. The size of terms represented the frequency of occurrence.

Figure 11 shows the 25 strongest citation bursts. Keywords “optical coherence tomography angiography,” “retinitis pigmentosa,” and “internal limiting membrane” burst in the last 3 years. Figure 12 shows the timeline for the evolution of keywords over 30 years. Figure 13 concludes the keywords of microperimetry research by wordcloud.

## 4. Discussion

In the present analysis, a total of 1,217 documents related to microperimetry from 1992 to 2022 were identified through the SCI-Expanded database in WOS. As an important research index, the number of academic documents can indicate the development directions in a research field.

The annual publication amount rose steadily in the past 30 years, especially in the last 10 years. It suggested the rapid development of the microperimetry technique and its wide application in the clinical practice of ophthalmology (11). In 1990, a scanning laser ophthalmoscope was utilized to detect precise retinal localization of the stimulus and fixation (12). The first microperimeter “MP-1” (Nidek Technologies, Padua, Italy) became available in 2003 (13). By adding a neutral density filter, the MP-1 microperimeter was modified to enhance scotopic sensitivity in 2011 (MP1-S) (14). In 2013, the MAIA microperimeter (CenterVue, Padua, Italy) was enrolled in the evaluation of macular function in retinal diseases (15). Therefore, the publication multiplied rapidly since 2013. In 2015, COMPASS fundus-guided perimeter (CenterVue, Padua, Italy) was developed to evaluate early-stage glaucoma with improved test repeatability (16). In 2016, the MP-3 microperimeter was introduced in detecting retinitis pigmentosa (17). MP-3 microperimeter had a wider range of threshold to overcome the ceiling effect in MP-1. The S-MAIA device was also developed to detect scotopic macular function in 2017 (18).

Original research articles accounted for 95.97%. It suggested that microperimetry is connected closely to ophthalmological clinical practice. Recent studies indicated that macular function, especially under mesopic or scotopic conditions, drew increasing attention in recent years (19, 20).

In co-authorship analysis, there existed a “clustering phenomenon.” That is to say, authors tended to collaborate with a relatively fixed partner. For instance, Herrmann P and Holz F collaborated on quite a few research. Similarly, Crossland M, alongside Rubin G, has also co-authored several articles. Authors were prone to cooperate with others in the same organization or academy. Co-operation between different institutes is difficult because of the inconsistency of equipment. The most productive authors were broadly located in Germany, the UK, Australia, and Italy. It indicated that microperimetry devices were accepted first in Europe. Country-wise, the USA, European countries, Australia, Japan, and China conducted extensive research. Univ Bonn, UCL, and Moorfields Eye Hosp introduced the technology of microperimetry earlier than other organizations and translated it to more scientific output.

There were a lot of journals that published high-quality articles about the utilization of microperimetry in ophthalmology. Retina-The Journal of Retinal and Vitreous Diseases, Investigative Ophthalmology and Visual Science, and American Journal of Ophthalmology were the top journals about ophthalmological research.

In keyword analysis, a transition of hotspots in microperimetry was presented. Interestingly, structural OCT measurements have frequently been shown to complement microperimetric functional testing in the studies. It was a precise morphological test that supplements microperimetry. In trend analysis, we found that in the 1990s, microperimetric testing was mainly aimed at the evaluation of visual outcomes after surgery of macular holes. In the 2000s, the hotspot of microperimetry changed to estimate the outcomes of macular degeneration and the effect of laser photocoagulation and photodynamic therapy for macular disease. During this time, fixation patterns and scotoma size had become new parameters for visual function. It was pointed out that central large scotomas developed worse than ring-shape ones (21). The size of the scotoma and reading speed may influence reading ability (22). In the 2010s, the mainstream of microperimetry usage was to evaluate the macular visual function in new clinical studies and trials, such as age-related macular degeneration, diabetic retinopathy, Stargardt’s disease, and retinitis pigmentosa (11). In addition, the relationship between structural and functional changes received great attention. Ultrahigh-resolution imaging with adaptive optics-optical coherence tomography was also introduced into multimodal analysis along with microperimetry (23). After 2020, OCT angiography technology became widely used in conjunction with microperimetry (24, 25). In retinitis pigmentosa, visual function and morphological changes were found to be associated with choriocapillaris defects but not with middle/large choroidal vascular defects (26). The inner retinal layers were closely associated with the functional integrity of the posterior pole (27). ILM-peeling was considered a controversial issue. Early studies showed that ILM-peeling damaged macular function and cause scotoma by ILM-peeling (28). However, by high-accuracy apparatus, recent research suggested that macular function did not decrease after ILM-peeling (29), even improved (30). With the sprouting of gene therapy, microperimetry shows potential for degenerative diseases in the future (31).

In cluster analysis, the term “central serous chorioretinopathy,” “fixation stability,” “fundus autofluorescence,” and “dark adaption” were clustered. Central serous chorioretinopathy was described as early as 1866, but the mechanisms were too complicated to elucidate clearly (32). As a result, the assessment of central serous chorioretinopathy needed the newly exact evaluation tools. Fixation pattern detection was another advantage of microperimetry (33). Fundus autofluorescence was also a complement to microperimetry (34, 35). Furthermore, microperimeters could not only evaluate the visual acuity but also help the rehabilitation training of fixation stability (34–36). The use of microperimetric fundus-tracking could be employed to potentially facilitate subtle changes that might be overlooked with standard perimetric examinations. Scotopic and mesopic test abilities were the crucial update of microperimetry instruments to fit the demands of dark adaption measurements (37).

There were several limitations of the study. First, the research focused on the authors and articles with the most citations. The authors of Africa and Latin America were not fully considered. Second, we paid close attention to only the topic and keywords. But the arguments of each topic were also worth analyzing. Finally, articles and research in the press might be included as well.

## 5. Conclusion

Microperimetry has developed for decades. It changed the concepts of traditional visual field examination and provides functional assessment combined with morphology. The bibliometric analysis of research status and trends in microperimetry provided global researchers with valuable information to propose future cooperation and track cutting-edge progress.

## Data availability statement

The original contributions presented in this study are included in the article/supplementary material, further inquiries can be directed to the corresponding author.

## Author contributions

JM designed the research, downloaded and analyzed the data, and wrote the manuscript. RQ edited and finalized the manuscript. Both authors contributed to the article and approved the submitted version.
